# Comparative Analysis of Early Follow‐up of Biologic Fixation and Cemented Stem Fixation for Femoral Tumor Prosthesis

**DOI:** 10.1111/os.12483

**Published:** 2019-06-26

**Authors:** Yuan Li, Yang Sun, Hua‐chao Shan, Xiao‐hui Niu

**Affiliations:** ^1^ Department of Orthopaedic Oncology Surgery, Beijing Ji Shui Tan Hospital Peking University Beijing China

**Keywords:** Biological fixation, Distal femoral tumor prosthesis, Prosthesis survival rate

## Abstract

**Objective:**

To compare the safety and efficacy between biologic fixation and traditional cement stems for the fixation of distal femoral prostheses for reconstruction following tumor resection.

**Methods:**

Retrospective analysis was performed of patients who received a first distal femoral tumor prosthesis, with a rotating hinge, in the Department of Orthopaedic Oncology of Beijing Jishuitan Hospital between October 2011 and January 2016. Two hundred and sixty eligible cases were enrolled, with a cemented fixation used in 199 of these cases and a biologic fixation in 61 cases. Survival rates and survival time of prostheses were analyzed, with prosthetic failure considered as the endpoint event for survival time of the prosthesis. Kaplan–Meier survival curve and the log‐rank test were used to compare survival rates between the two types of fixation methods, and factors that may affect the survival rate of prosthesis were evaluated.

**Results:**

Of the 260 cases forming our study group, 138 were males and 122 females, with 102 males and 97 females in the cemented fixation group (mean age, 25.8 years; range, 8–72 years) and 36 males and 25 females in the biologic fixation group (mean age, 25.5 years; range, 12–59 years). Osteosarcoma was the most common type of tumor (188 cases, 72.3%), of which 145 cases (72.9%) were in the cemented and 45 cases (72.1%) in the biologic fixation group. Among the 260 cases enrolled into the study group, 13 patients were lost to follow‐up. The average duration of follow‐up for the remaining 247 cases was 28.8 months (median, 28.8 months; range, 4–61 months). The 3‐year overall survival rate of prostheses was 87.2% for the biologic fixation group and 80.4% in the cemented fixation group (*P* = 0.389). The 3‐year mechanical survival rate (excluding cases of infection and oncologic progression) was 100% for the biologic fixation and 97.6% for the cemented fixation group (*P* = 0.468). Complications were identified in 21 cases: 3 cases (5%) in the biologic and 18 cases (9.6%) in the cemented fixation group (*P* = 0.264). Two revisions were required in the cemented fixation group, but no revision was required in the biologic fixation group. A total of 10 patients required amputation after prosthesis implantation. Of these, 7 cases (4 cement and 3 biologic) were due to tumor recurrence; 3 cases were due to infection, with all cases occurring in the cement fixation group.

**Conclusion:**

The current study provides a baseline reference for future mid‐term to long‐term follow‐up, laying the foundation for further studies and comparison of the incidence of aseptic loosening of both types of prosthesis.

## Introduction

With advances in adjuvant therapy, surgical techniques and prosthetic design and materials, limb salvage surgery has become the main treatment for malignant or invasive bone tumors of the limbs^1^, ^2^, ^3^. The distal femur is a common site for bone tumors, with a distal femoral tumor prosthesis typically used for reconstruction after tumor resection^4^, ^5^. The advantages of a tumor prosthesis include good early stage reliability, rapid postoperative recovery, better cosmetic effect, improved psychological acceptance by patients, and satisfactory limb function. However, the mid‐to‐long term rate of complications, such as infection, mechanical weathering, and aseptic loosening, often lead to the eventual failure of the prosthetic implants^6^, ^7^. In their review of 2861 patients treated with a distal femoral tumor prosthesis, Henderson *et al*. reported aseptic loosening to be the most important complication, with an incidence rate of 11.5% and accounting for 43.1% of all complications^8^.

Aseptic loosening as the primary cause of the long‐term failure of distal femoral tumor prosthesis failure has been confirmed in other studies, with the incidence of loosening of the tibial component of the prosthesis being very low^8^, ^9^, ^10^. The current intramedullary fixations for distal femoral tumor prosthesis are divided into two types: cemented fixation and biologic fixation^6^, ^7^. For cemented fixation, cement was used to bulk‐fill the space between the prosthesis and the bone and to form a microscopic mechanical interlocking between the prosthesis and the bone, providing an instant fixation. Bone cement is a chemical polymerization agent, having an elastic modulus that is between that of cancellous bone and the metal of the prosthesis. After initial fixation, the long‐term stability of the prosthesis depends on maintenance of a cement‐to‐bone interlocking interface, the quality of the fixation, and the strength of the cement itself. Bone cement is susceptible to fatigue fracture under long‐term stress, generating cement microparticles that lead to periprosthetic osteolysis, causing prosthesis loosening. The biologic fixation (LINK Megasystem‐C) prosthesis used consisted of titanium alloy body and stem, with microspores and hydroxyapatite (HA) coating on the surface of the biologic stem. The biologic fixation was rendered through two stages, a mechanical stage and a biological stage. For the initial mechanical fixation, the medullary cavity was ground and extended in strict accordance with the perimeter and length of the prosthesis. All cancellous bone without strong fixation capacity was removed, allowing the prosthesis to be closely integrated with the medullary cavity for fixation. During the biologic fixation stage, the close match between the prosthesis and the medullary cavity allows bone growth and ossification to be tightly integrated with the prosthesis, with both the HA coating and titanium alloy integrating tightly with the surrounding bone, through both chemical bonds and biological binding. This enhances the binding force at the interface between the prosthesis and the medullary cavity. The biologic fixation achieves the binding of the prosthesis with the medial cortical bone, increasing the long‐term axial and rotational stability of the prosthesis. The coated collar promotes the formation extracortical bone bridge, thereby improving stress transmission and closing the prosthesis–bone joint to avoid wear particles entering the prosthesis–bone interface, and theoretically solves the problems of the traditional cemented fixation. Cemented fixation of the prosthesis was used in all these studies, with the efficacy of biologic fixation on prosthesis survival remaining to be clarified.

Therefore, the aim of our study was to compare the safety and efficacy of femoral reconstruction between a biologic and conventional cemented fixation of the distal femoral component of a tumor prosthesis. The biologic fixation used in our center is the LINK Megasystem‐C prosthesis, which is the only biologic fixation of tumor prosthesis approved by the China Food and Drug Administration (CFDA). The control group used the cemented fixation, which has been well accepted technically and therapeutically. The two groups of patients in this study received treatments during the same period of time in our medical center. Retrospective analysis was performed on cases who received distal femoral tumor prosthetic replacement in our department from October 2011 to January 2016. The survival rate and survival time of prostheses were analyzed, with prosthetic failure considered as the endpoint event for survival time of the prosthesis. Oncological progression, amputation, and death were also analyzed. The Kaplan–Meier survival curve and the log‐rank test were used to compare survival rates between the two types of fixation methods, and factors that may affect the survival rate of prosthesis were evaluated.

We expected the biologically fixed distal femoral tumor prosthesis used in this study to show no significant difference in the safety and efficacy of early stage compared with the cemented fixed prosthesis. Hence, that this result could be consistent with the design theory and expectations of biologic prostheses. Of course, the original intention of the design of biologic fixation is to solve the problem of long‐term aseptic loosening of cemented fixation. The long‐term follow‐up results of these patients deserve close clinical attention. Therefore, we hope that this report will: (i) provide a baseline and standard for future mid‐term to long‐term follow‐up; (ii) laying the foundation for further studies; and (iii) enable comparison of the incidence of aseptic loosening of both types of prostheses and their influencing factors.

## Materials and Methods

### *Participants*

Retrospective analysis was performed of patients who received a prosthesis for a distal femoral tumor in the Department of Orthopedic Oncology of Beijing Jishuitan Hospital between October 2011 and January 2016. Clinical, oncologic, and imaging data were extracted from the patient database in our department. Included in our study were patients who received a first distal femoral tumor prosthesis, with a rotating hinge. Patients who underwent revision implantation or in whom a simple hinge prosthesis was not used were excluded. We identified 260 eligible cases, with a cemented fixation used in 199 of these cases and a biologic fixation in 61 cases.

### *Interventions*

#### Biologic Fixation

**Figure 1:**
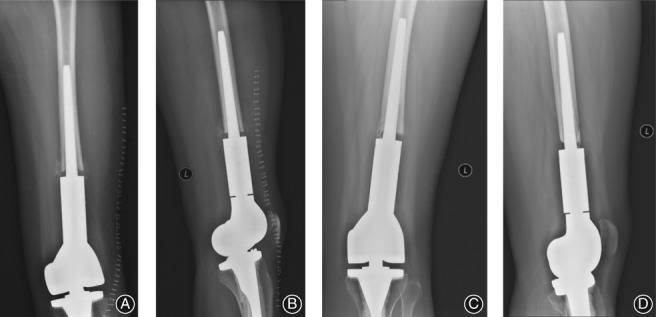
Biologic fixation of a distal femoral prosthesis in a 24‐year‐old woman following resection of an osteosarcoma. Frontal (A) and lateral (B) radiographs showing an adequate contact between the prosthetic stem and the medial side of the cortical bone in the medullary cavity, with a visible 1–2 mm space between the prosthesis stem and the bone. Repeat frontal (C) and lateral (D) radiographs obtained 54 months post‐implantation, showing good contact between the prosthesis and the medullary cavity and satisfactory fixation, with the space between the bone and the prosthesis being maintained, with formation of a bony bridge.

The biologic fixation (LINK Mega system‐C) prosthesis used consisted of titanium alloy body and stem, with microspores and hydroxyapatite coating on the surface of the biologic stem. For biologic fixation, a bur was used to manually expand the medullary cavity to the required depth; electric expansion was not used to avoid excessive expansion. The size of the medullary cavity was gradually increased until the contact between the conical medullary cavity bur and the cortical bone was ≥50 mm. The biologic stem and the medullary cavity bur stem were matched, and the prosthesis stem was connected to the stem holder and placed into the femoral medullary cavity until immobilized by pressure. The appropriate positioning of the stem within the cavity was obtained by applying even force on the stem holder using a small hammer. A residual space of 1–2 mm was left between the bone and the prosthesis to prevent formation of an incomplete contact. During the surgery, the stem holder could be rotated to test the stability of the prosthesis. If the femur rotates simultaneously with the prosthesis, then effective fixation can be confirmed. Otherwise, if the size of the prosthesis is too small, a prosthesis one size larger should be used. If the femoral fracture occurred during the installation of the prosthetic stem, a titanium cable can be used for fixation across the fractured region. The purpose of coating the collar of the prosthesis is to allow bone growth over the junction between the cortical bone and the prosthesis to form a cortical bone bridge. This extracortical bone bridge can theoretically enhance prosthesis fixation, improving stress transmission through a close contact surface between the prosthesis and the bone to avoid wear particles entering the prosthesis–bone interface^11^, ^12^, ^13^ (Fig. 1).

#### Cemented Fixation

For the cemented fixation, we used the standard technique which has evolved with the first generation of cement in the 1970s to the current third generation, including thorough expansion of medullary cavity, a cement gun to introduce the cement into the cavity, use of a large‐diameter prosthesis stem, and centralization of the prosthesis stem within the medullary cavity to ensure a uniform distribution of bone cement. An ideal bone cement thickness of approximately 2 mm was used, with a thinner layer likely to cause bone cement fracture^10^. After initial fixation, the long‐term stability of the prosthesis depends on maintenance of a cement‐to‐bone interlocking interface, the quality of the fixation, and the strength of the cement itself. Weakness of any of these components will lead to overall failure^10^, ^13^, ^14^.

### *Comparisons*

The following data were extracted for analysis: age, sex, diagnosis, side of reconstruction, follow‐up time, surgical and prosthetic complications, prosthetic survival, prosthetic loosening, reason for prosthetic failure, treatment regimen after prosthetic failure, tumor recurrence, metastasis, the Musculoskeletal Tumor Society (MSTS) score^15^, and patient survival.

#### Causes of Prosthetic Failure

Using the classification by Henderson *et al*.^8^, the following 5 causes of prosthetic failure were considered: (i) soft tissue failure (joint instability, tendon tear and aseptic wound dehiscence); (ii) aseptic loosening; (iii) prosthetic failure (periprosthetic fracture or prosthesis breaking); (iv) infection; and (v) tumor progression.

#### Location of Prosthetic Loosening

**Figure 2:**
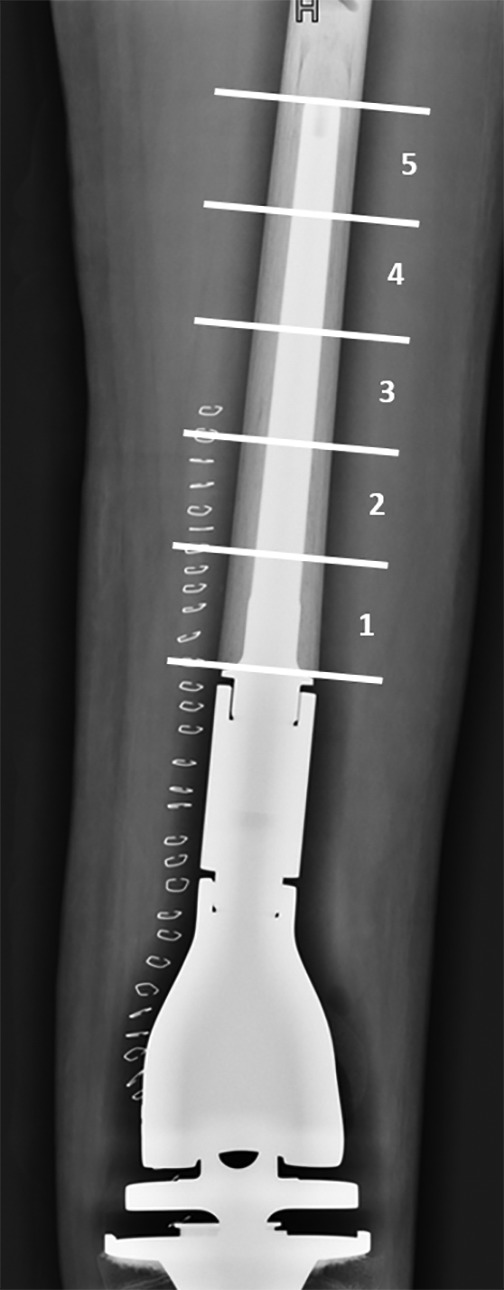
Radiographs of the stem of the distal femoral prosthesis (analysis zones).

The location of prosthetic loosening was defined according to the method previously described by Shah *et al*.^16^: On plain radiographs, the prosthesis stem was divided into 5 equal zones, with each zone corresponding to 20% of the total length of the prosthesis stem, with zone 1 starting from the junction between the body and the stem of the prosthesis and zone 5 ending at the endpoint of the prosthesis stem (Fig. 2). The appearance of translucent bands from zone 1 to zone 3 during the follow‐up period was recorded as clinically stable prosthesis loosening.

### *Statistical Analysis*

The survival rate and survival time of prosthesis were analyzed, with prosthetic failure considered as the endpoint event for survival time of the prosthesis. Oncological progression, amputation, and death were also analyzed. SPSS19.0 statistical software was used. Categorical variables were described by their frequency and percentage, with continuous variables described by their mean, range and median value. Kaplan–Meier survival curve and the log‐rank test were used to compare survival rates between the two types of fixation methods, and factors that may affect the survival rate of prosthesis were evaluated.

## Results

### *Overview*

#### Demographics

Of the 260 cases forming our study group, 138 were men and 122 women, with 102 men and 97 women in the cemented fixation group (mean age, 25.8 years; range, 8–72 years) and 36 men and 25 women in the biologic fixation group (mean age, 25.5 years; range, 12–59 years). Osteosarcoma was the most common type of tumor (188 cases, 72.3%), of which 145 cases (72.9%) were in the cemented and 45 cases (72.1%) in the biologic fixation group. Giant cell tumor of bone was the second most common tumor type (34 cases, 13.1%), of which 24 cases were in the cemented (12.1%) and 10 cases (16.4%) in the biologic fixation group. Other tumors included chondrosarcoma (8 cases), pleomorphic undifferentiated sarcoma (7 cases), spindle cell sarcoma (6 cases), Ewing sarcoma (4 cases), malignant giant cell tumor of bone (3 cases), and miscellaneous types of tumors (9 cases). There was no statistically significant difference in age, sex, and diagnosis distributions between the two groups.

#### Length of Osteotomy

The average length of the osteotomy was 163.1 cm (median, 160 cm; range, 80–320 cm) for the cemented fixation group and 154.4 cm (median 150 cm; range, 100–305 cm) for the biologic fixation group. The prosthesis stem length was 143.1 cm (median, 150 cm; range, 80–180 cm) for the cemented fixation group and 135.4 cm (median, 130 cm; range, 100–160 cm) for the biologic fixation group. There was no statistically significant difference in the length of osteotomy and the length of prosthesis between the two groups.

#### Follow‐up Time

Among the 260 cases enrolled into the study group, 13 patients were lost to follow‐up. The average duration of follow‐up for the remaining 247 cases was 28.8 months (median, 28.8 months; range, 4–61 months). For the 187 cases in the cemented fixation group, the average follow‐up was 29.1 months (median, 28.2 months; range, 4–59 months), with an average follow‐up of 27.7 months (median, 23.6 months; range, 6–61 months) for the 60 cases in the biologic fixation group. There was no statistically significant difference in the duration of follow‐up between the two groups. The prosthesis‐related outcomes are summarized in Table 1, with the types of failures summarized in Table 2.

**Table 1:** Prosthesis‐related outcomes for the 247 cases

Prosthesis‐related events	Total	Total percentage (%)	Cement fixation group	Percentage in the cement fixation group (%)	Biologic fixation group	Percentage in the biologic fixation group (%)	*P*‐value
Survival of prosthesis without events	208	84.21	154	82.35	54	90.00	0.62
Revision (mechanical failure)	2	0.81	2	1.07	0	0.00	0.17
Amputation after recurrence	7	2.83	4	2.14	3	5.00	0.24
No amputation after recurrence	5	2.02	5	2.67	0	0.00	0.25
Amputation after infection	3	1.21	3	1.60	0	0.00	0.29
No amputation after infection	3	1.21	2	1.07	1	1.67	0.64
Death	19	7.69	17	9.09	2	3.33	0.24
Total	247	100	187	100	60	100	

**Table 2:** Types of prosthesis failure, according to the criteria of Henderson *et al*.

Prosthesis failure type	Total	Total percentage (%)	Cement fixation group	Percentage in the cement fixation group (%)	Biologic fixation group	Percentage in the biologic fixation group	*P*‐value
Soft tissue failures	0	0	0	0	0	0	—
Aseptic loosening	0	0	0	0	0	0	—
Structural failures	2	0.81	2	1.07	0	0.00	0.17
Infection	3	1.21	3	1.60	0	0.00	0.29
Tumor progression	7	2.83	4	2.14	3	5.00	0.24
Total	12	4.86	9	4.81	3	5.00	0.95

### *Prosthesis Survival*

**Figure 3:**
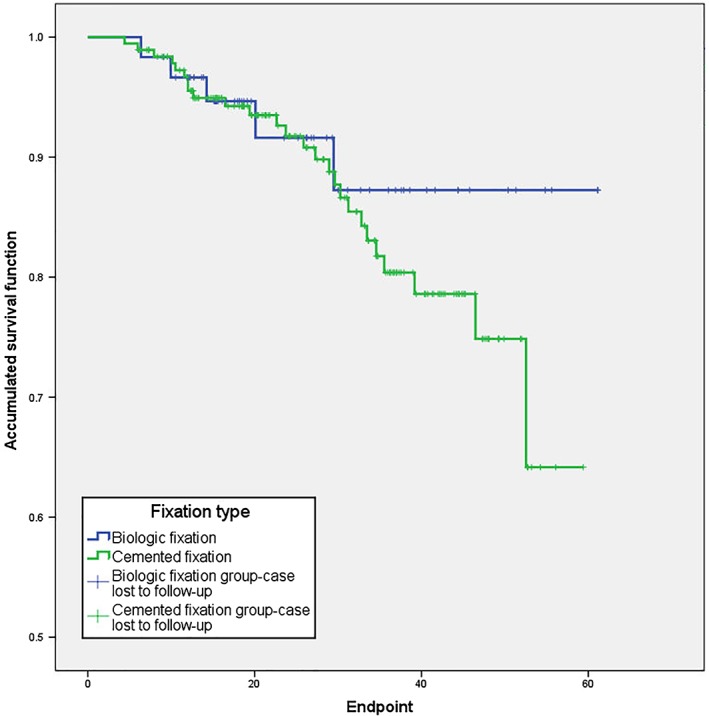
Overall survival rate of prosthesis.

**Figure 4:**
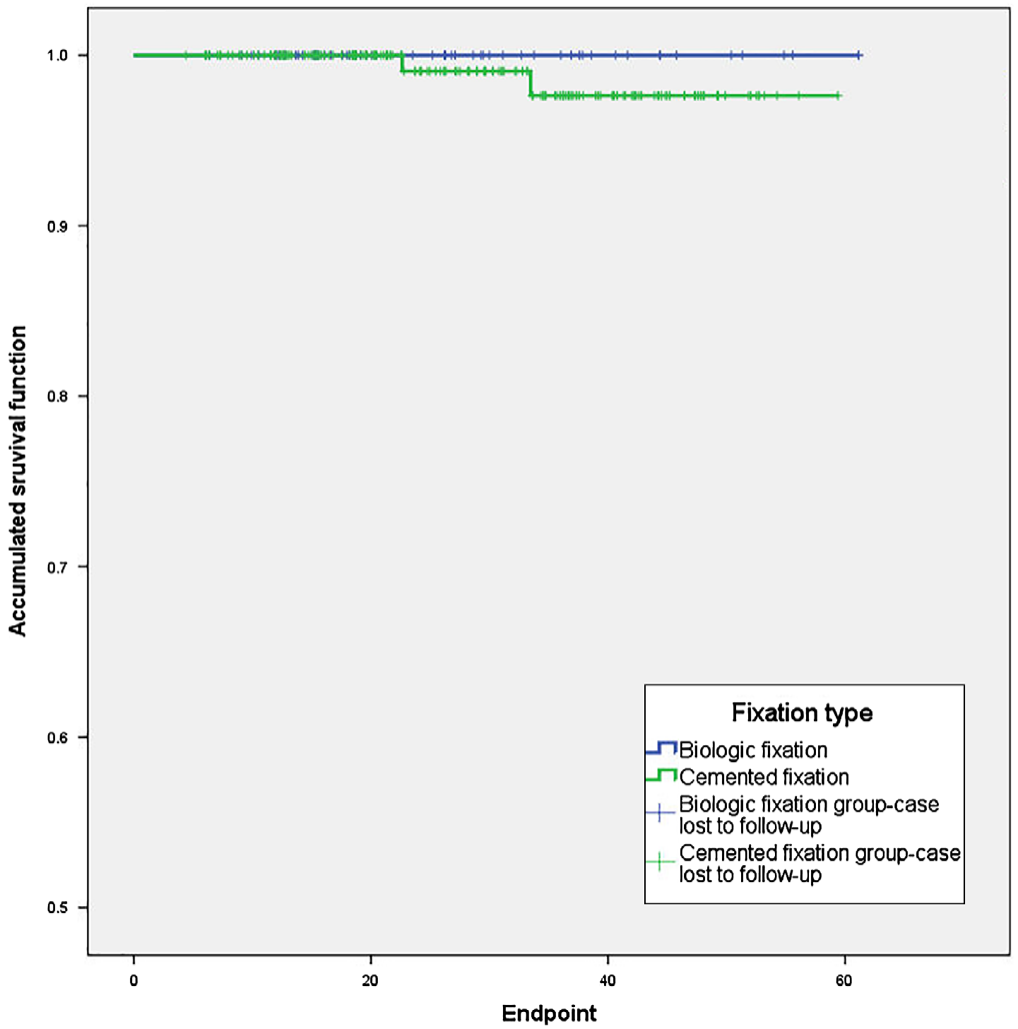
Mechanical survival rate of prosthesis.

The overall survival rate of prostheses and the mechanical survival rates are shown in Figs 3 and 4, respectively. The 3‐year overall survival rate was 87.2% with biologic fixation and 80.4% with a cemented fixation (*P* = 0.389). The 3‐year mechanical survival rate (excluding cases of infection and oncological progression) was 100% and 97.6%, respectively, for the biologic and cemented fixation (*P* = 0.468).

### *Complications*

Overall, complications were identified in 21 cases, 3 cases (5%) in the biologic and 18 cases (9.6%) in the cemented fixation group (*P* = 0.264). Complications in the biologic fixation group included: 1 case of deep infection treated by surgical debridement, irrigation, and drainage, and 2 cases of delayed wound healing, treated by debridement. Complication in the cemented fixation group included: 5 cases of infection, 3 of which required amputation, with the other 2 cases successfully treated conservatively; 6 cases of delayed wound healing, with 4 of these cases treated using wound dressing and 2 by debridement; 2 cases of peroneal nerve injury, 1 case resulting in temporary impairments and the other to permanent injury; 3 cases of intraoperative vascular injury, with 1 case of femoral vein involvement treated with ligation and 2 cases of femoral artery injury requiring intraoperative repair, with none of these cases developing serious vascular‐related complications; 1 case of postoperative hematoma, which was treated symptomatically; and 1 case of leg length discrepancy, due to a pre‐existing flexion deformity. In the cemented fixation group, 2 patients sustained an open prosthetic injury caused by a fall, which was combined with a patellar fracture in 1 case. Both patients were treated with debridement and suture, with no subsequent prosthetic infection or loosening over the course of follow‐up.

#### Revision and Amputation

With regard to the outcomes of the prosthesis, two revisions were required in the cemented fixation group due to fracture of the prosthesis stem at 23 months post‐implantation in 1 patient and at 33 months in the other. Both fractures occurred in zone 1 of the prosthesis, with no other instance of prosthesis problems in these patients up to the endpoint of follow up. No revisions were required in the biologic fixation group, with no between‐group difference (*P* = 0.468).

Overall, 10 patients required amputation after prosthesis implantation. Of these, 7 cases were due to tumor recurrence, which occurred on average 16 months (median, 11 months; range, 5–56 months) after prosthesis implantation, 4 cases in the cement and 3 in the biologic fixation group (*P* = 0.24). Amputation was required in the remaining 3 cases due to infection, with all cases occurring in the cement fixation group at 12, 39, and 53 months after prosthesis implantation. This incidence rate, however, was not significant (*P* = 0.287).

#### Aseptic Loosening

**Figure 5:**
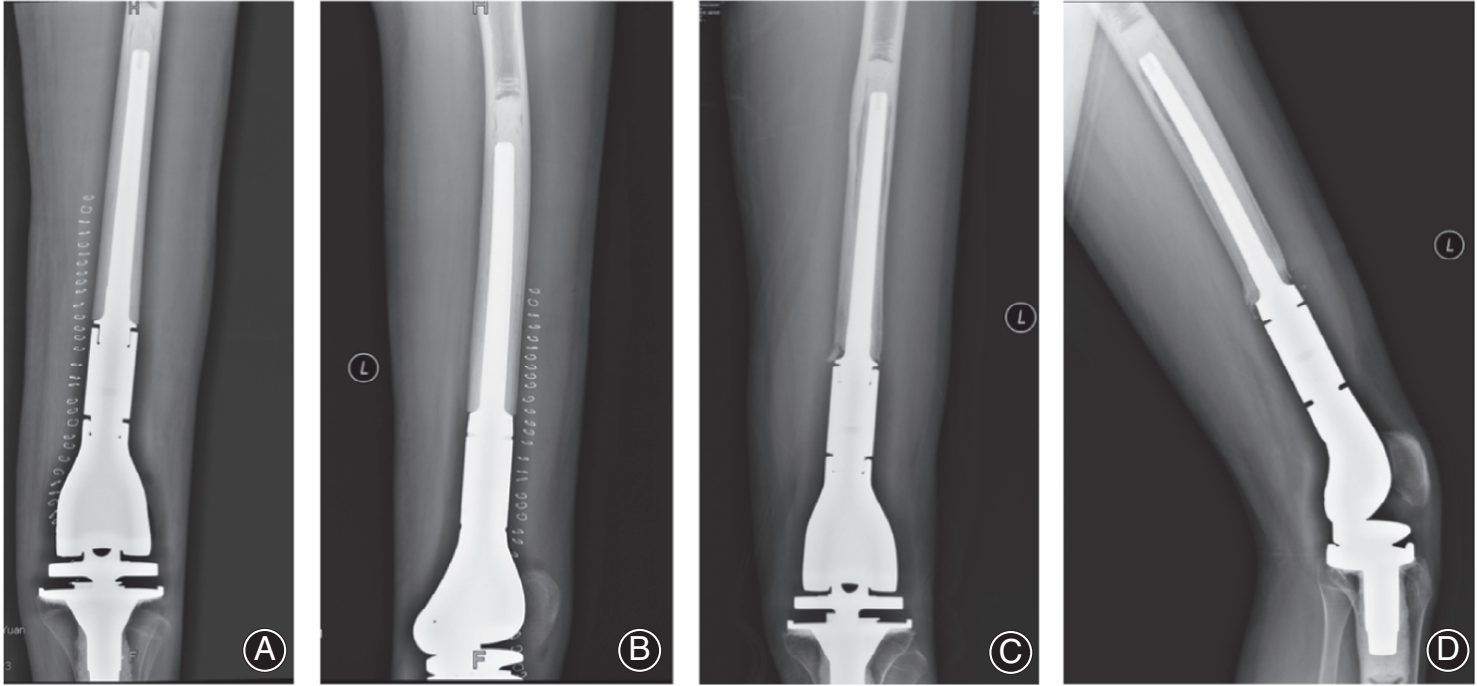
Aseptic loosening of the distal femoral component in a 14‐year‐old male treated for an osteosarcoma using cemented fixation of the distal prosthesis. Frontal (A) and lateral (B) views of the distal femoral component after surgery show a centralized positioning of the stem, with a translucent band visible between the cement layer of the proximal prosthesis stem and the cortical bone. Frontal (C) and lateral (D) view of the distal femur obtained 37 months after the surgery, with the translucent band being larger than on the initial baseline radiographs obtained post‐surgery.

**Figure 6:**
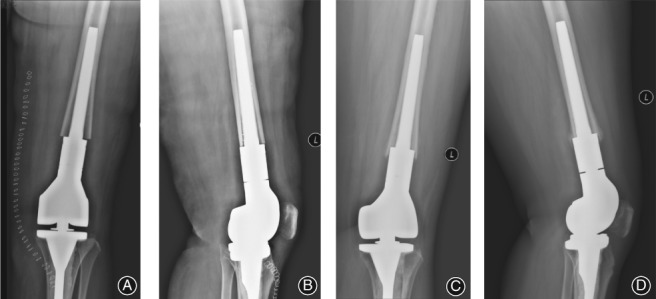
Aseptic loosening in a 40‐year‐old woman treated for a chondrosarcoma, using biologic fixation of the prosthesis. Frontal (A) and lateral (B) views of the distal femur, obtained after surgery, with a short contact length between the stem and the cortical bone of the medullary cavity, with absence of a 3‐point fixation and no visible space between the stem and the medullary cavity. Frontal (C) and lateral (D) view of the distal femur obtained 8 months after prosthesis implantation. The prosthesis stem could not form an effective fixation with the medullary cavity, with upward migration of the prosthesis and the patient complaining of pain.

With regard to aseptic loosening of the prosthesis, which excludes cases due to infection, translucent bands were identified in 5 cases in the cement fixation group and 1 case in the biologic fixation group. In the cement group, translucent bands were identified in zones 1–2 in 1 case, zones 1–3 in 3 cases and in zones 1–5 in 1 case. None of the patients complained of pain; however, for the case with a translucent band in zones 1–5, the prosthesis migrated upwards, with a shortening >2 cm of the involved leg (Fig. 5). A similar upward migration of the prosthesis was identified for the case in the biologic fixation group, 2 years after implantation, with the patient presenting with pain and shortening of the involved leg (Fig. 6). None of the cases of aseptic loosening required revision, and there was no instance of loosening of the tibial component.

The two groups of patients in this study received treatments during the same period of time in our medical center, and the age and diagnosis distribution were similar to those in published studies; and there is no statistical difference between the two groups. The difference in the early stage oncological factor‐caused or infection‐caused prosthetic failure between the two groups is not statistically significant (Table 2). Although there was no incidence of aseptic loosening in either group, structural failure due to a fracture close to the prosthesis stem occurred in the cemented fixation group.

In our study, we evaluated stem loosening based on the imaging criteria defined by Shah *et al*.^16^, and according to the proposal by Bergin *et al*.^10^ that the translucent bands through zones 1–3 are indicative of aseptic loosening. In our study group, translucent bands were identified in zones 1–3 in 3 cases, in zones 1–2 in 1 case and in zones 1–5 in 1 case, the latter being associated with upward migration of the prosthesis (>2 cm) and consequent shortening of the limb. By contrast, translucent bands were identified in only 1 case in the biologic fixation group, which was associated with an upward migration, despite formation of a cortical bridge at the junction of the prosthesis and bone, and limb shortening.

## Discussion

### *Aseptic Loosening*

Aseptic loosening is the most important cause of distal femoral tumor prosthesis failure and the most common long‐term complication^6^, ^8^, ^9^, ^17^, and may differ for the two main types of intramedullary fixation methods; namely, cemented and biologic fixation. The current intramedullary fixations for distal femoral tumor prosthesis are divided into two types: cemented fixation and biologic fixation^6^, ^7^, ^18^, ^19^. Bone cement is susceptible to fatigue fracture under long‐term stress, generating cement microparticles that lead to periprosthetic osteolysis, causing prosthesis loosening^20^. By contrast, biologic fixation binds the prosthesis to the medial cortical bone, increasing the long‐term axial and rotational stability of the prosthesis. Moreover, the coated collar promotes the formation of an extracortical bone bridge, which improves stress transmission and closes the prosthesis–bone joint to avoid wear particles entering the prosthesis–bone interface and, theoretically, solves the problems of traditional cemented fixation^12^, ^21^, ^22^. In their review of the Compress biologic fixation prosthesis, Pedtke *et al*.^13^ reported a rate of aseptic loosening of 3.8%, compared to 11.5% for a cemented fixation, over a 6‐year follow‐up period. Farfalli *et al*.^12^ reported a comparable 5‐year prosthesis survival rate for two types of biologic fixation, the fixed press‐fit and Compress distal femoral prosthesis. However, there is no comparison in the application of press‐fit biologic fixation and cemented fixation in the distal femoral tumor prosthesis.

The stability of biologic fixed knee joint prostheses depends on achievement of a good press‐fit between the bone and the prosthesis. This fit is limited by a patient's bone condition and the accuracy requirement for osteotomy. According to a meta‐analysis, the prosthetic dislocation of the biologically fixed prosthesis at 2 years after the surface replacement surgery of the knee joint was significantly higher than that of the cemented fixation^23^. However, Daniilidis *et al*.^24^ believe that the degree and extent of loosening of biologic tibial prostheses were significantly higher than those of cemented fixed prostheses. Therefore, the patients in this study all used cemented fixed prostheses for tibial surface replacement.

As cemented fixation is well accepted, both from a technical and therapeutic perspective, we used cemented fixation as the control group in our study. Moreover, prostheses in both groups were implanted during the same period of time at our medical center, with the two groups balanced for age and diagnosis. Therefore, our study provides a reliable comparison of outcomes between biologic and cemented fixation prosthesis.

Although there was no incidence of aseptic loosening in either group, structural failure due to a fracture close to the prosthesis stem occurred in the cemented fixation group, with no evidence of loosening in zones 2–5. Our rate of structural failure was 1.08% lower than previously reported rates of 3.8%–6.3%^8^, ^19^, which might reflect our limited follow‐up duration and/or improvement of cemented fixation techniques. Bhangu *et al*.^19^ used the same short‐term follow up, 2 years on average, with a reported rate of structural failure of 3.8% (1/26 cases) for cemented fixation and 7.7% (2/26 cases) for biologic fixation, with no incidence of aseptic loosening. In their study of 16 cases of rotating hinge cemented prosthesis, Shih *et al*.^25^ reported a failure rate of 6% (1/16 cases) over an average follow up of 2.3 years, again with no incidence of aseptic loosening. In their series of 9 cases followed over an average of 1.5 years, Freedman *et al*.^26^ reported 1 case of aseptic loosening (11%) and 1 case of infection (11%). Therefore, over the first 2 years post‐implantation, the probability of aseptic loosening is low, regardless of whether a cemented or biologic fixation is used, with non‐oncological prosthesis failures mainly resulting from structural failure and prosthetic infection.

There is evidence that the rate of aseptic loosening does increase over longer durations of follow up. In their 3‐year follow‐up of 251 biologically fixed prostheses, Mittermayer *et al*.^17^ reported a rate of aseptic loosening of 8.4%, with Unwin *et al*.^9^ reporting a rate of 9.9% over a 4‐year follow‐up and Kawai *et al*.^27^ reporting a rate of 27.5% for 40 cases with an average follow‐up of 4.25 years. Although the current concept of bone cemented fixation has progressed, including the use of bone cement guns, fully medullary extension and the use of prosthetic stems with larger diameters, which reduces the early loosening of cemented fixed prosthesis, the factors causing aseptic loosening have not been resolved. Therefore, the cases in the cement group in this study may present similar loosening rates as those previously reported over a longer follow‐up period.

Studies with follow‐up periods >5 years have been conducted to evaluate the survival rate of tumor prosthesis. In a case series of 52 distal femoral tumor prosthesis, Pedtke *et al*.^13^ reported a rate of aseptic loosening of 11.5% for cemented prosthesis compared to 3.8% for the Compress biologically fixed prosthesis over 6 years, although this difference in rate was not significant. Jeys *et al*.^5^ followed 228 patients over 9.3 years, reporting a rate of aseptic loosening of 13.6%. Mittermayer *et al*.^28^ and Unwin *et al*.^9^ reported a 10‐year rate of aseptic loosening of 24% and 32.6%, respectively.

In our study, we evaluated stem loosening based on the imaging criteria defined by Shah *et al*.^16^, and according to the proposal by Bergin *et al*.^10^ that the translucent bands through zones 1–3 are indicative of aseptic loosening. In our study group, translucent bands were identified in zones 1–3 in 3 cases, in zones 1–2 in 1 case and in zones 1–5 in 1 case, the latter being associated with upward migration of the prosthesis (>2 cm) and consequent shortening of the limb. Although none of these patients complained of pain, close clinical monitoring is warranted. By contrast, translucent bands were identified in only 1 case in the biologic fixation group, which was associated with an upward migration, despite formation of a cortical bridge at the junction of the prosthesis and bone, and limb shortening. Factors likely to have contributed to an insufficient bone–prosthesis interface included: absence of a space between the prosthesis and the bone at the end of the osteotomy (0 mm compared to a mean of 1.2 mm for this group); absence of an effective 3‐point fixation; and a short contact length between the prosthesis stem and the medial cortical bone (45 mm compared to an average 72.9 mm for this group). Of note, these factors were identified in another 3 cases in which the prosthesis remained stable over the follow‐up period. It is likely that these cases will also progress to loosening due to resorption of the bone ends, bone thickening, and formation of bone bridges caused by changes in the stress distribution at the bone–prosthesis interface. Therefore, a longer follow‐up duration is needed to fully characterize the rate of aseptic loosening of biologically fixed prostheses.

The indications for biologic prosthesis include: reconstruction of a large bone deficit following tumor resection; prosthesis revision; and trauma. Contraindications include: local or systemic acute and chronic infection; allergy to the implant material; effective contact surface <80 mm; and bone loss that is sufficient to limit prosthetic stability. Relative contraindications include: obesity; muscular, vascular, and neurological disorders that may pose a threat to the involved limb; and conditions that may cause excessive loading on the prosthesis or stress accumulation^12^, ^13^, ^19^, ^20^, ^21^, ^22^, ^29^.

### *Other Complications*

In our study, the 2‐year limb salvage rate was comparable in both groups: 96.3% for cemented and 95% for biologic fixation, rates that were consistent with early limb salvage rates previously reported. These rates are comparable to those reported by Bhangu *et al*.^19^ over a 2‐year follow‐up, of 92.3% for cemented and 96% for biologic fixation, with 6‐year rates of 88.5% for both fixation types^13^. Similarly, Kawai *et al*.^27^ reported a 3‐year limb salvage rate of 93%, and a 5‐year rate of 90%.

Infections, caused by diverse and complex factors, are serious complications of tumor prosthesis which are difficult to control. Our infection rate was 2.7% in the cemented and 1.7% in the biological fixation group. By comparison, Henderson *et al*.^8^ reported a rate of 5.4% among 2861 cases of distal femoral tumor prosthesis. Our lower rate of infection could result from our relatively short follow‐up period, and the possibly of an increase in the rate over time cannot be excluded. Once a prosthetic infection occurs, it is generally difficult to control and cure, especially in patients with defective immunity due to chemotherapy, and amputation is usually the final outcome.

### *Limitations*

Our findings are limited by the retrospective nature of our study, the relatively short follow‐up time, and the choice of prosthesis which was generally influenced by economic factors. Randomized, prospective studies are warranted to confirm findings.

### *Summary*

In summary, over an average of 29 months of follow up, there were no differences in the early rate of prosthesis survival and prosthesis‐related events between the cemented and biologic fixation groups, with comparable short‐term safety and efficacy for both fixation methods, a finding consistent with previous reports. Of course, the original intention of the design of biologic fixation is to solve the problem of long‐term aseptic loosening of cemented fixation. Although not significant, there was a higher incidence of the development of translucent lines (indicative of possible aseptic loosening) in the cemented than the biologic fixation group. However, long‐term follow‐up would be needed to clarify the clinical significance of these differences. Therefore, our report provides a baseline reference for future mid‐to‐long term follow‐up, laying the foundation for further studies and comparison of the incidence of aseptic loosening of both types of prosthesis and of factors influencing outcomes.
